# Management of psoriatic arthritis: a consensus opinion by expert rheumatologists

**DOI:** 10.3389/fmed.2023.1327931

**Published:** 2023-11-30

**Authors:** Salvatore D’Angelo, Fabiola Atzeni, Maurizio Benucci, Gerolamo Bianchi, Fabrizio Cantini, Roberto Felice Caporali, Giorgio Carlino, Francesco Caso, Alberto Cauli, Francesco Ciccia, Maria Antonietta D’Agostino, Lorenzo Dagna, Christian Dejaco, Oscar Massimiliano Epis, Maria Grazia Ferrucci, Franco Franceschini, Enrico Fusaro, Marco Gabini, Roberto Gerli, Roberto Giacomelli, Marcello Govoni, Elisa Gremese, Giuliana Guggino, Annamaria Iagnocco, Florenzo Iannone, Bruno Laganà, Ennio Lubrano, Carlomaurizio Montecucco, Rosario Peluso, Roberta Ramonda, Maurizio Rossini, Carlo Salvarani, Gian Domenico Sebastiani, Marco Sebastiani, Carlo Selmi, Enrico Tirri, Antonio Marchesoni

**Affiliations:** ^1^Rheumatology Department of Lucania, San Carlo Hospital of Potenza, Potenza, Italy; ^2^Rheumatology Unit, Department of Experimental and Internal Medicine, University of Messina, Messina, Italy; ^3^Rheumatology Unit, S. Giovanni di Dio Hospital, Florence, Italy; ^4^Division of Rheumatology, Department of Medical Specialties, Azienda Sanitaria Locale 3 Genovese, Genova, Italy; ^5^Private Practice, Prato, Italy; ^6^Division of Clinical Rheumatology, ASST Gaetano Pini-CTO Institute, Milan, Italy; ^7^Department of Clinical Sciences and Community Health, University of Milan, Milan, Italy; ^8^Rheumatology Service, ASL LE-DSS Casarano and Gallipoli, Gallipoli, Italy; ^9^Department of Clinical Medicine and Surgery, University of Naples Federico II, Naples, Italy; ^10^Rheumatology Unit, Department of Medicine and Public Health, AOU and University of Cagliari, Cagliari, Italy; ^11^Rheumatology Section, Department of Precision Medicine, University of Campania "Luigi Vanvitelli", Naples, Italy; ^12^Department of Rheumatology, Catholic University of Sacred Heart, Fondazione Policlinico Universitario A. Gemelli, IRCSS, Rome, Italy; ^13^Unit of Immunology, Rheumatology, Allergy and Rare Diseases (UnIRAR), San Raffaele Scientific Institute, Milan, Italy; ^14^School of Medicine, Vita-Salute San Raffaele University, Milan, Italy; ^15^Department of Rheumatology and Immunology, Medical University of Graz, Graz, Austria; ^16^Department of Rheumatology, Teaching Hospital of the Paracelsius Medical University, Brunico Hospital (ASAA-SABES), Brunico, Italy; ^17^Division of Rheumatology, Multispecialist Medical Department, ASST Grande Ospedale Metropolitano Niguarda, Milan, Italy; ^18^Department of Rheumatology, Azienda Ospedaliera Rummo Benevento, Benevento, Italy; ^19^Rheumatology and Clinical Immunology Unit, Dipartimento Continuità di Cure e Fragilità, ASST Spedali Civili di Brescia, Brescia, Italy; ^20^Department of Clinical and Experimental Sciences, University of Brescia, Brescia, Italy; ^21^Rheumatology Unit, University Hospital AOU Città della Salute e della Scienza di Torino, Turin, Italy; ^22^Rheumatology Unit, Santo Spirito Hospital, Pescara, Italy; ^23^Rheumatology Unit, Department of Medicine and Surgery, University of Perugia, Perugia, Italy; ^24^Research Unit of Immuno-Rheumatology, Department of Medicine, School of Medicine, University of Rome "Campus Biomedico", Rome, Italy; ^25^Fondazione Policlinico Campus Bio-Medico, Rome, Italy; ^26^Rheumatology Unit, Department of Medical Sciences, Azienda Ospedaliero-Universitaria S. Anna-Ferrara, University of Ferrara, Ferrara, Italy; ^27^Clinical Immunology Unit, Department of Geriatrics, Orthopedics and Rheumatology, Fondazione Policlinico Universitario A. Gemelli-IRCCS, Catholic University of the Sacred Heart, Rome, Italy; ^28^PROMISE, Università degli studi di Palermo, Palermo, Italy; ^29^Academic Rheumatology Centre, Department of Clinical and Biological Sciences, Università degli Studi di Torino, Turin, Italy; ^30^DiMePRe-J, Rheumatology Unit, Università degli studi di Bari “Aldo Moro”, Bari, Italy; ^31^Department of Clinical and Molecular Medicine, Sapienza University of Rome-S. Andrea University Hospital, Rome, Italy; ^32^Academic Rheumatology Unit, Department of Medicine and Health Sciences "Vincenzo Tiberio", Università Degli Studi del Molise, Campobasso, Italy; ^33^Department of Internal Medicine and Therapeutics, Rheumatology Unit, University of Pavia, IRCCS Policlinico S. Matteo, Pavia, Italy; ^34^Department of Clinical Medicine and Surgery, School of Medicine, University Federico II of Naples, Naples, Italy; ^35^Rheumatology Unit+ EULAR Center of Excellence in Rheumatology, Department of Medicine-DIMED, University of Padova, Padua, Italy; ^36^Rheumatology Unit, Department of Medicine, University of Verona, Verona, Italy; ^37^Azienda USL-IRCCS di Reggio Emilia, Università di Modena e Reggio Emilia, Reggio Emilia, Italy; ^38^Rheumatology, Azienda Ospedaliera San Camillo Forlanini, Rome, Italy; ^39^Rheumatology Unit, CHIMOMO, University of Modena and Reggio Emilia, Modena, Italy; ^40^Department of Biomedical Sciences, Humanitas University, Pieve Emanuele, Italy; ^41^Department of Rheumatology and Clinical Immunology, Humanitas Clinical and Research Center-IRCCS, Rozzano, Italy; ^42^Rheumatology Unit, Ospedale del Mare, Naples, Italy; ^43^Rheumatology, Humanitas San Pio X, Milan, Italy; ^44^Ospedale S. Maria Nuova, Reggio Emilia, Italy

**Keywords:** psoriatic arthritis, chronic inflammatory musculoskeletal disease, comorbidities, extra-articular manifestations, diagnosis, treatment, consensus process, expert opinion

## Abstract

**Background:**

Psoriatic arthritis (PsA) is a chronic inflammatory musculoskeletal disease involving several articular and extra-articular structures. Despite the important progresses recently made in all of the aspects of this disease, its management is still burdened by unresolved issues. The aim of this exercise was to provide a set of statements that may be helpful for the management of PsA.

**Methods:**

A group of 38 Italian rheumatologists with recognized expertise in PsA selected and addressed the following four topics: “early PsA,” “axial-PsA,” “extra-articular manifestations and comorbidities,” “therapeutic goals.” Relevant articles from the literature (2016–2022) were selected by the experts based on a PubMed search. A number of statements for each topic were elaborated.

**Results:**

Ninety-four articles were selected and evaluated, 68 out of the 1,114 yielded by the literature search and 26 added by the Authors. Each of the four topic was subdivided in themes as follows: transition from psoriasis to PsA, imaging vs. CASPAR criteria in early diagnosis, early treatment for “early PsA”; axial-PsA vs. axialspondyloarthritis, diagnosis, clinical evaluation, treatment, standard radiography vs. magnetic resonance imaging for “axial PsA”; influence of inflammatory bowel disease on the therapeutic choice, cardiovascular comorbidity, bone damage, risk of infection for “comorbidities and extra-articular manifestations”; target and tools, treat-to-target strategy, role of imaging for “therapeutic goals.” The final document consisted of 49 statements.

**Discussion:**

The final product of this exercise is a set of statements concerning the main issues of PsA management offering an expert opinion for some unmet needs of this complex disease.

## 1 Introduction

Psoriatic arthritis (PsA) is a chronic inflammatory musculoskeletal disease associated with psoriasis, or a predisposition to this skin disorder, which may involve joints, entheses, and the axial skeleton. In addition, PsA may be associated with extra-articular manifestations such as inflammatory bowel disease (IBD) and uveitis, and with a number of comorbidities, first of all those metabolic in nature.

Articular and extra-articular manifestations, as well as comorbidities, may have a profound impact on the quality of life of patients with PsA and may even be responsible for a shorter life expectancy (1–5). Early diagnosis, comprehensive disease assessment, and proper treatment are the mainstays to guarantee the best outcome of PsA patients, both in the short and long-term. In the past two decades, relevant research progresses have been made in understanding pathophysiology and defining clinical phenotypes (6–11), and therapies targeting new mechanisms of action have been developed (12). Despite these improvements, the management of PsA is still difficult and many unresolved questions in this field await an answer (13).

To provide a guidance in this complex topic, a group of Italian rheumatologists with expertise in PsA (Expert Group: EG) convened to elaborate, through a consensus process, a number of statements addressing some of the main issues of diagnosis, assessment, and treatment of PsA.

## 2 Methods

Thirty-eight Italian rheumatologists with leading roles and expert in PsA agreed to participate to this consensus study. The expert group was composed on the basis of the following criteria:

- Clinical experience in psoriatic arthritis management.
- Research activity in psoriatic arthritis disease.
- Participation in disease-specific guidelines and scientific committees, indicating a commitment to improving standards of care and promoting best practices in disease management.
- Participation in conferences and congresses as a speaker, demonstrating commitment to the scientific community and the opportunity to share the latest findings and establish collaborative links.

Fifteen of them constituted a steering committee which selected, among several “hot” general topics in the management of PsA considered of interest, the following four for their relevance: early PsA, axial PsA, comorbidities and extra-articular manifestations, and therapeutic goals. The process was then structured in subsequent steps.

In the firststep, the steering committee explored the main issues concerning the four selected topics, evaluated a literature review previously performed, and defined the specific items to be addressed. The literature review was carried out by an independent methodologist in the Medline via PubMed using as searching definition “psoriatic arthritis AND early,” “psoriatic arthritis AND axial,” “psoriatic arthritis AND comorbidity,” “psoriatic arthritis AND extraarticular manifestations,” and “psoriatic arthritis AND therapy.” Only references in English and published within January 1st, 2016 and December 31st, 2021 were selected. The methodologist performed a first screening of the retrieved records by title and summary and excluded all those not relevant to the search question. Duplicates were marked to be removed from the final manuscript count but left for evaluation by any individual subgroups (see below). The remaining records were evaluated by the steering committee, which selected only the manuscript considered of interest. The final selection was then forwarded to the EG, which was subdivided into four subgroups, one for each of the topics previously defined by the steering committee. Finally, as the various consensus rounds were eventually held in 2022, manuscripts published in 2022 and considered of relevance by the components of the EG were also included in the literature evaluation.

For the second step, each of the four subgroups convened online to discuss the themes of interest and elaborate a number of statements relevant to any individual theme. These statements were then evaluated through a Delphi-like process (14). Each of them was voted by the components of the steering committee using a 9-point scale (ranging from 1, strongly disagree to 9, strongly agree). Then, median scores were calculated for each statement: a median score greater than or equal to 7 was considered a positive consensus, between 3 and 7 a neutral opinion, and lower than 3 a negative consensus. Individual responses were anonymous to preserve objectivity. The statements with a negative consensus were discussed, modified if needed, and then voted again until an agreement was reached. The final product for each working group was a document containing the approved statements.

In the third step, the final four documents were submitted for anonymous evaluation to the entire panel of participating rheumatologists and each statement was scored as reported above.

## 3 Results

**Figure 1:**
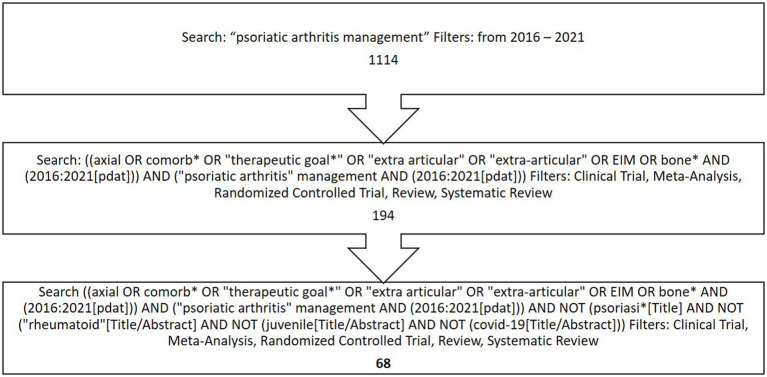
Flowchart describing the literature selection process.

The steering committee selected and evaluated 68 of the 1,114 articles originally yielded by the literature search, to which were added another 26 manuscripts published in 2022 and considered relevant to the various topics (Figure 1).

After the various rounds, the final document consisted of 49 statements subdivided as follows: 11 for “early PsA” (Table 1A), 12 for “axial-PsA” (Table 1B), 19 for “comorbidities and extra-articular manifestations” (Table 1C), and 7 for “therapeutic goals” (Table 1D). The themes of interest explored by the EG were the following:

- for “early PsA”: transition from psoriasis to PsA, imaging vs. CASPAR criteria in early diagnosis, and early treatment.
- for “axial PsA”: axial-PsA vs. axial-spondyloarthritis (SpA), diagnosis, clinical evaluation, treatment, and standard radiography vs. MRI.
- for “comorbidities and extra-articular manifestations”: influence of IBD on the therapeutic choice, cardiovascular comorbidity, bone damage, and risk of infection.
- for “therapeutic goals”: target and tools, treat-to-target (T2T) strategy, and role of imaging.

**Table 1A:** Statements stemming from the discussion of the topic “Early PsA” and the score, out of a 9-point scale, they received from the consensus group.

	Early PsA [ref. (15–40)]		
N.	Statements	Median score	Consensus achieved
	**Transition from psoriasis to PsA**		
1	A biomolecular approach could contribute in the future, together with clinical and imaging factors, to identify patients at risk of transitioning to PsA	7.80	YES
2	Different cellular subsets of innate immunity (mucosal associated invariant T cells, innate lymphoid cells, γδ-T cells, tissue resident memory cells) currently seem to guide the pathogenesis of psoriasis and PsA. Further studies are needed to support their role in the transition from psoriasis to PsA	7.87	YES
3	The IL-17/IL-23 axis and its cytokines (IL-17, IL-22 and IL-23) hierarchically is the most relevant axis in the pathogenesis of psoriasis and PsA. However, the phenotypic heterogeneity of psoriatic disease requires further studies to define whether this concept can be applicable to all patients	7.90	YES
4	To date, the available literature data are not enough to clarify whether the treatment of psoriatic patients with biological drugs can modify the probability of evolution toward PsA	7.87	YES
	**PsA diagnosis: imaging vs. CASPAR classification criteria**		
5	Given the lack of biomarkers and the need for early PsA diagnosis in patients with psoriasis, it is important to enhance the training of dermatologists in diagnostics	7.90	YES
6	Ultrasound (US) alone is not decisive for differentiating PsA from other rheumatological diseases, but in some cases may help	7.74	YES
7	Both magnetic resonance (MRI) and US are useful for the early and accurate assessment of inflammation and damage in the PsA	7.93	YES
8	Imaging may facilitate early disease classification by integrating CASPAR criteria	7.74	YES
9	Imaging (US and MRI) provides information useful to the analysis of the subclinical forms to support the diagnosis and to predict the future course of the disease	7.25	YES
	**Early treatment**		
10	Although there is no agreement in the literature on when bone damage begins and on the correlation between blockade of inflammation and damage progression, it is considered appropriate to immediately begin the proper treatment for the manifestations of the disease and its comorbidities	8.32	YES
11	It is essential to intercept PsA patients with oligosymptomatic disease. To this end, collaboration with dermatologists and GPs is essential in order to promptly refer to the rheumatologist the patients with psoriasis and symptoms of possible osteo-articular involvement.	8.64	YES

**Table 1B:** Statements stemming from the discussion of the topic “Axial PsA” and the score, out of a 9-point scale, they received from the consensus group.

	Axial PsA [ref. (41–63)]		
N.	Statements	Median score	Consensus achieved
	**Axial-PsA vs.-axial SpA**		
12	Axial-PsA and axial-SpA represent two different clinical entities	7.45	YES
13	The diagnosis of the axial form of PsA is based on the use of imaging	7.12	YES
14	To date, the prevalence (and incidence) of axial-PsA is still difficult to define. Depending on the definition used, the reported axial involvement ranges from 25 to 70%. In patients with early PsA the prevalence ranges from 5 to 28%. The radiographic evolution increases with the duration of the disease and is very frequently associated with peripheral involvement (only 2–5% of PsA patients have a purely axial form)	7.40	YES
15	Imaging-only classification may lead to overestimation, as some patients may have DISH or edema due to mechanical stress or overload (patients may be elderly, overweight)	7.40	YES
16	Inflammatory back pain must always be considered as a clinical element for a correct identification of axial involvement	7.50	YES
	**Clinical evaluation**		
17	The tools generally used for the assessment of the peripheral disease are not useful for the purpose of evaluation of the axial involvement of PsA	7.50	YES
18	Due to the lack of dedicated indices, the tools generally used for the assessment of axial-SpA can also be used for the assessment of the axial involvement of PsA	7.20	YES
19	The INSPIRE study concluded that BASDAI could be used for the assessment of axial involvement in PsA but was influenced by the simultaneous presence of peripheral involvement. This is also supported by some reviews, which suggests to consider only the BASDAI questions relating to axial involvement. The ASAS-EULAR recommendations, updated in June 2022, however abolished the BASDAI as an evaluation index of the axial-PsA. It is, therefore, agreed that the tools used for the assessment of axial-SpA (especially ASDAS) can also be used for the evaluation of the axial involvement of PsA, taking into account that the peripheral involvement may influence also this index. However, it is emphasized that the tools available are inadequate	7.20	YES
	**Treatment**		
20	Inhibition of IL23 could be a treatment target for axial-PsA, although to date there is no evidence of efficacy in axial SpA. Although IL23 is fundamental from a pathogenetic point of view in SpA, the inhibition of IL-23 has not reached the primary outcome in studies of axial-SpA	7.10	YES
	**Imaging: standard radiography vs. MRI/TC**		
21	A pelvic and a spine x-ray should always be performed in patients with a history of inflammatory axial pain newly diagnosed with PsA. Evidence on the pelvis x-ray of a unilateral grade 2 sacroiliitis is sufficient to make a diagnosis of axial-PsA	7.15	YES
22	In case of negative pelvic X-ray in a patient with inflammatory axial pain and psoriasis or psoriatic arthritis, MRI of the sacroiliac joints should always be performed with dedicated sequences.	7.93	YES
23	There is no agreement on the need to perform diagnostic imaging in asymptomatic patients. It is proposed to limit the radiography of the pelvis in a PsA patient in two cases: (1) history of doubtful inflammatory or mechanical low back pain or (2) the need to guide the therapeutic decision	7.36	YES

**Table 1C:** Statements stemming from the discussion of the topic “Comorbidities and extra-articular manifestations” and the score, out of a 9-point scale, they received from the consensus group.

	Comorbidities and extra-articular manifestations [ref. (64–84)]		
N.	Statements	Median score	Consensus achieved
	**Inflammatory bowel disease (IBD): influence on the therapeutic choice**		
24	Three patient profiles are to consider: 1. IBD patients in remission 2. Patients with active IBD 3. Patients with new onset IBD	7.60	YES
25	Therapeutic choice may be influenced by the presence of subclinical intestinal inflammation detected by fecal calprotectin values>100 mcg/gr or by a previous colonoscopy, once other possible causes of elevated calprotectin values have been excluded. Colonoscopy only to detect subclinical intestinal inflammation is not recommended.	8.03	YES
26	Measurement of fecal calprotectin at baseline also appears to be advisable in patients with PsA.	8.16	YES
27	If a patient is undergoing treatment with DMARDs for intestinal disease and requires therapy for arthritis, it is important to assess the compatibility of the new therapy with the ongoing treatment. If the treatments are not compatible, it is necessary to consult a gastroenterologist to explore alternative therapy options for the intestinal disease.	7.93	YES
28	If the patient is not on DMARD therapy for bowel disease and a therapy for arthritis is required, the therapy can be started, preferably using a drug also indicated for the treatment of bowel disease.	8.09	YES
29	In patients with active IBD and PsA needing treatment, the consultation of the gastroenterologist is necessary to formulate the prescription, given the higher dosage usually required for the treatment of IBD.	7.00	YES
30	In patients with new-onset IBD and PsA under treatment, the therapeutic choice will be conditioned by the PsA ongoing therapy. In case of treatment with MTX monotherapy, etanercept or anti IL-17, the therapy will need to be changed.	8.35	YES
	**Cardiovascular comorbidity**		
31	Cardiovascular comorbidity is important in PsA. PsA patients are twice more likely than the general population to develop a heart condition. A correlation between inflammation of the enthesis and cardiovascular involvement has recently been demonstrated.	7.87	YES
32	Both inflammation and metabolic syndrome play a relevant role in the cardiovascular risk of PsA.	8.16	YES
33	The evaluation of cardiovascular risk scores and screening with laboratory tests and method with non-invasive diagnostic imaging, are recommended in patients with PsA. It is advisable to evaluate the patient’s risk score at baseline and then periodically. Second level screening is suggested for patients with more severe disease and higher cardiovascular risk. There was agreement on the need to identify risk categories in which to stratify patients. Those with greater risk should be directed to a more in-depth analysis, possibly with an investigation of the vascular tree (ultrasound of the supra-aortic vessels). The cardiovascular risk should be evaluated using specific scores (e.g., Framingham’s score), assessing disease activity (as defined by the DAPSA), and defining type and severity of articular involvement.	7.87	YES
34	Data in PsA are lacking, but in rheumatoid arthritis stable remission reduces the risk of ischemic cardiovascular events by 53%. The goal, therefore, is to define the best possible treatment, in order to prevent both the joint and cardiovascular manifestations	8.16	YES
	**Bone damage**		
35	Structural damage, including bone damage, has a significant and irreversible impact on physical function and on the disability of the PsA patient	8.32	YES
36	Erosive lesions associated with enthesophytes are characteristic, though not exclusive, of PsA.	7.83	YES
37	In PsA, and already in psoriasis, there are early microstructural alterations of the bone, both at cortical and at trabecular level.	7.83	YES
38	The qualitative and quantitative alterations of the bone occur both focally and systemically, with an increased risk of osteoporosis and fragility fractures.	7.58	YES
39	Conventional radiography can be useful for the diagnosis and the follow up of PsA, since the presence of erosions is predictive of damage progression.	7.74	YES
40	There is currently little and contradictory evidence on the predictors of radiographic progression. At present, the only predictors of radiographic progression in PsA remain increased values of the CRP and a previous structural damage.	7.58	YES
	**Risk of infection**		
41	PsA patients have an increased infectious risk compared to psoriasis patients and the general population. Infectious risk must be assessed based on the following favoring factors: Use of glucocorticoids, immunosuppressive drugs and combination therapies High disease activity Presence of comorbidities (this is one of the reasons for the greater frequency of infections in elderly patients).	8.00	YES
42	In patients at high risk of infection it is preferable to indicate a pharmacological strategy other than TNFα blockade. Anti-TNFα drugs should also be avoided in patients with chronic obstructive pulmonary disease and with a previous history of tuberculosis. While anti TNFα drugs increase the risk of bacterial infections, using the inhibition of the IL17 the greatest risk is fungal. Although the literature data are still few, the results of clinical trials suggest that inhibitors of the IL12/IL23 pathway and selective inhibitors of IL23 are associated with a lower risk of developing infection than anti-TNFα drugs.	7.51	YES

**Table 1D:** Statements stemming from the discussion of the topic “Therapeutic goals” and the score, out of a 9-point scale, they received from the consensus group.

	Therapeutic goals [ref. (85–102)]		
N.	Statements	Median score	Consensus achieved
	**Targets and tools**		
43	The unique characteristics of this disease make it difficult to use a single metric index that would be sufficient for evaluating each individual patient. However, utilizing different indices separately can provide a more accurate identification of the prevalent disease domains. It’s important to take into account the evaluation of experts who can determine which index or indices to use based on the prevalence of specific manifestations.	7.93	YES
44	This task force, and EULAR guidelines for treatment, recommend that remission should be the main goal, or low disease activity as an alternative, although there is no ideal tool to define the target.	8.06	YES
	**Treat-to-target strategy (T2T)**		
45	The cross referral (when necessary) between rheumatologists and dermatologists is of paramount importance in the early identification of patients and for the most correct definition of the therapeutic target in the individual patient.	8.00	YES
46	Modern treatment of PsA involves identifying the various subsets of the disease and making decisions in collaboration with the patient. This process requires considering the patient’s perspective, as well as properly weighing the patient-reported outcomes that are a part of their overall experience. This is functional to adherence to therapy.	8.09	YES
47	It is essential to make a correct diagnosis as early as possible and to define the timing of treatment and follow up	8.16	YES
48	There is a lack of shared indications and evidence in the literature on how (and if) to modify the treatment in a patient in remission	8.16	YES
	**Role of imaging**		
49	The various imaging methods are useful within the respective disease domains, both in diagnosis and in the evaluation of clinical outcome and anatomical damage. The application of ultrasonography in the T2T strategy remains to be evaluated	7.87	YES

All of the themes of interest and relative approved statements are reported in Table 1. Results can be summarized for each specific theme as follows: (i) dermatologists are important in detecting PsA and early diagnosis and treatment are likely to be of great benefit, (ii) there are large knowledge gaps in the distinction between axial-PsA and axial-SpA, on how to diagnose axial PsA, its prevalence, how to assess it, and how to treat it; (iii) associated conditions and comorbidities of major clinical relevance include IBD, cardiovascular and metabolic disturbances and bone damage; and (iv) measuring treatment targets in PsA should be based on instruments more suitable for the clinical manifestations of each individual patient.

## 4 Discussion

The purpose of this consensus study was to provide an expert opinion on some issues concerning the management of patients with PsA. The choice of the topics to be addressed was arbitrarily made by the steering committee of the EG. Many other themes would have been of interest, but the four selected subjects were considered among the most relevant for the clinical management of PsA patients and are all included in the research agenda of the recent recommendations developed by EULAR (62) and GRAPPA (63).

The “early PsA” topic was mainly focused on the transition from psoriasis to PsA and early diagnosis and treatment. It was agreed that, despite the advances in the pathophysiology knowledge (6, 15–21), prediction of the transition at a molecular level is still not possible. Thus, the dermatologist ability to detect the psoriatic patients at risk of PsA should be enhanced as much as possible. Although ultrasound (US) imaging and magnetic resonance imaging (MRI) are not always specific for PsA, their use may help for its early diagnosis, classification and assessment (30–32). However, it is worth noting that the statement on the US and MRI imaging was the one with the lowest agreement (7.25) of this topic, indicating that the role of these imaging techniques in early PsA needs to be studied further. Finally, even if the evidence is scarce, it was underlined that early treatment of PsA may guarantee the best outcome, while more data are needed to prove that treating psoriatic patients with immunosuppressive drugs may prevent the transition to PsA (38–40). Overall, the EG agrees that that dermatologists play an important role in detecting PsA and that early diagnosis and treatment are likely to be of great benefit.

Globally, the topic “axial-PsA,” showed the lowest rate of agreement, with only one statement (“In case of negative pelvic X-ray in a patient with inflammatory axial pain and psoriasis or psoriatic arthritis, MRI of the sacroiliac joints should always be performed with dedicated sequences”) reaching a score of nearly 8. This result likely mirrors the well-known controversies concerning this theme. The first statement about this topic was that axial-PsA and axial-SpA likely represent two different entities. As the evidence on this subject is not conclusive, it could be argued that this statement only reflects personal opinions. However, based on genetic factors and clinical and radiographic findings, a growing number of experts in the field are supporting the concept that axial-PsA and axial-SpA cannot be considered the same entity (42–47). Many other questions on this topic remain unanswered: how to diagnose axial PsA, its prevalence, how to assess it, and how to treat it. As for the diagnosis, it was reasoned that diagnosis should be based on imaging, using radiography as first technique, which, however, should be performed only in symptomatic patients. Inflammatory back pain should always be sought for in patients with PsA. An imaging-driven diagnosis of axial PsA will exclude patients with axial involvement without radiographic or MRI changes. This choice was made to avoid the risk of diagnosing as axial PsA all psoriatic patients with back pain. In addition, it was considered not appropriate to perform axial imaging investigation in all patients, regardless of their symptoms. For the assessment of axial PsA, given the lack of specific instruments, it was indicated that the BASDAI and, preferably, the ASDAS may be used. Finally, for the treatment, as all of the recommendations clearly indicate that anti-TNF-α and IL-17 drugs are the therapy of choice for axial-PsA, only the issue recently arisen of the possible efficacy of anti-IL23 therapies on this disease domain was addressed. The final agreement was that ongoing studies should show whether anti-IL23 therapies are effective to treat axial-PsA (54–56).

The “comorbidity and extra-articular manifestations” topic addressed four themes: IBD, cardiovascular comorbidity, bone damage, and infection risk. As for the IBD, all the various possible clinical occurrences were analyzed. Basically, it was suggested that drugs effective for both the articular and the intestinal disease should be preferred in most cases, to be used with the co-operation of the gastroenterologist whenever needed. Interestingly, it was agreed that the measurement of fecal calprotectin may be advisable in patients with PsA. As there are no data to support this statement, it was only based on the experts’ opinion. It was reckoned that values of fecal calprotectin greater than 100 μg/gr in absence of other possible causes might be due to subclinical intestinal inflammation and thus should be considered for the therapy choice. For the cardiovascular (CV) comorbidity, it was stated that the CV risk should be scored using specific instruments in all patients with PsA and that subjects at elevated risk should undergo in-depth investigations (e.g., supra-aortic vessels ultrasound). Despite the lack of definite evidence, it was felt that proper management of the CV risk factors and of the articular disease should decrease the incidence of CV events. Bone damage was also included in the “comorbidity and extra-articular manifestations” section. The statements on this subject underlined the importance of evaluating the bone damage through standard radiography. Infections are undoubtedly a major concern when immunosuppressive agents are used for the treatment of PsA. It was stated that before starting a therapy, patients should be carefully evaluated particularly for the well-known risk factors of infection and treated accordingly. Although not definitive, the available data indicate that IL-17 and IL-23 inhibitors are less likely to favor bacterial infections than TNF-α blockers (81–85).

The fourth topic, “therapeutic goals,” recorded the highest level of agreement, with most statements reaching a score greater than 8. As indicated by all recent international recommendations (62, 63), remission, or at least a status of minimal disease activity, was considered the goal of the therapy. Given the phenotypic heterogeneity of PsA, it was suggested to assess the disease activity using the instruments more suitable for the clinical manifestations of each individual patient. It was emphasized the importance of cooperating with a dermatologist whenever needed and of considering the patient’s opinion to optimize the adherence. For a personalized T2T approach, it was indicated to define time of intervention and follow-up according to the individual clinical context. The available data on treatment modifications in case of remission were considered not strong enough to provide indications. This opinion is not in line with what indicated in the most recent recommendations on the treatment of PsA, which state that drug tapering, and eventually even drug discontinuation, may be considered in case of disease remission. The EG did not advise against this strategy, but decided that at present the available evidence dot not allow to draw definite conclusions on this issue (100–102). Finally, it was affirmed that imaging is useful to assess disease evolution, but a possible role of the US in a T2T strategy remains to be established.

## 5 Conclusion

We provide the results of an exercise which, moving from the available literature, resulted in statements on the understanding and management of PsA. The main limitations of this work are that that the period chosen is short and that not all potentially useful literature has been included to answer the research questions, and that the majority of statements are not based on definite evidence and only reflected the opinion of a group of experts, thus being liable to criticism. On the other hand, the purpose of this exercise was to provide an opinion on some unresolved questions regarding the management of PsA and Delphi-like methods are considered acceptable to provide indications when evidence is weak or absent. The choice of the topics to be addressed was arbitrary, yet there was large agreement on their relevance to the practicing rheumatologist.

## Data availability statement

The original contributions presented in the study are included in the article/supplementary material, further inquiries can be directed to the corresponding author.

## Author contributions

SD’A: Formal analysis, Supervision, Writing – original draft, Writing – review & editing. FA: Formal analysis, Supervision, Writing – review & editing. MB: Formal analysis, Writing – review & editing. GB: Formal analysis, Writing – review & editing. FCan: Formal analysis, Writing – review & editing. RC: Formal analysis, Supervision, Writing – review & editing. GC: Formal analysis, Writing – review & editing. FCas: Formal analysis, Writing – review & editing. AC: Formal analysis, Supervision, Writing – review & editing. FCi: Formal analysis, Supervision, Writing – review & editing. MD’A: Formal analysis, Supervision, Writing – review & editing. LD: Formal analysis, Writing – review & editing. CD: Formal analysis, Writing – review & editing. OE: Formal analysis, Writing – review & editing. MF: Formal analysis, Writing – review & editing. FF: Formal analysis, Writing – review & editing. EF: Formal analysis, Writing – review & editing. MGa: Formal analysis, Writing – review & editing. RGe: Formal analysis, Writing – review & editing. RGi: Formal analysis, Supervision, Writing – review & editing. MGo: Formal analysis, Writing – review & editing. EG: Formal analysis, Writing – review & editing. GG: Formal analysis, Writing – review & editing. AI: Formal analysis, Supervision, Writing – review & editing. FI: Formal analysis, Supervision, Writing – review & editing. BL: Formal analysis, Writing – review & editing. EL: Formal analysis, Supervision, Writing – review & editing. CM: Formal analysis, Writing – review & editing. RP: Formal analysis, Writing – review & editing. RR: Formal analysis, Supervision, Writing – review & editing. MR: Formal analysis, Supervision, Writing – review & editing. CSa: Formal analysis, Supervision, Writing – review & editing. GS: Formal analysis, Writing – review & editing. MS: Formal analysis, Writing – review & editing. CSe: Formal analysis, Supervision, Writing – review & editing. ET: Formal analysis, Writing – review & editing. AM: Formal analysis, Supervision, Writing – original draft, Writing – review & editing.
